# Transposon-mediated insertional mutagenesis unmasks recessive insecticide resistance in the aphid *Myzus persicae*

**DOI:** 10.1073/pnas.2100559118

**Published:** 2021-05-31

**Authors:** Michela Panini, Olga Chiesa, Bartlomiej J. Troczka, Mark Mallott, Gian Carlo Manicardi, Stefano Cassanelli, Filippo Cominelli, Alex Hayward, Emanuele Mazzoni, Chris Bass

**Affiliations:** ^a^Department of Sustainable Crop Production, Section Sustainable Crop and Food Protection, Università Cattolica del Sacro Cuore, 29122, Piacenza, Italy;; ^b^College of Life and Environmental Sciences, Biosciences, University of Exeter, Cornwall TR10 9FE, United Kingdom;; ^c^Dipartimento di Scienze della Vita, Università di Modena e Reggio Emilia, 42122 Modena, Italy

**Keywords:** transposon, resistance, voltage-gated sodium channel

## Abstract

The evolution of resistance to insecticides threatens the sustainable control of many of the world’s most damaging insect crop pests and disease vectors. To effectively combat resistance, it is important to understand its underlying genetic architecture, including the type and number of genetic variants affecting resistance and their interactions with each other and the environment. While significant progress has been made in characterizing the individual genes or mutations leading to resistance, our understanding of how genetic variants interact to influence its phenotypic expression remains poor. Here, we uncover a mechanism of insecticide resistance resulting from transposon-mediated insertional mutagenesis of a genetically dominant but insecticide-susceptible allele that enables the adaptive potential of a previously unavailable recessive resistance allele to be unlocked. Specifically, we identify clones of the aphid pest *Myzus persicae* that carry a resistant allele of the essential voltage-gated sodium channel (VGSC) gene with the recessive M918T and L1014F resistance mutations, in combination with an allele lacking these mutations but carrying a Mutator-like element transposon insertion that disrupts the coding sequence of the VGSC. This results in the down-regulation of the dominant susceptible allele and monoallelic expression of the recessive resistant allele, rendering the clones resistant to the insecticide bifenthrin. These findings are a powerful example of how transposable elements can provide a source of evolutionary potential that can be revealed by environmental and genetic perturbation, with applied implications for the control of highly damaging insect pests.

## Significance

The ability to control damaging plant pests and vectors of animal disease is threatened by the emergence of insecticide resistance. Developing effective strategies to prevent, slow, or overcome resistance requires an understanding of the underlying genetic mechanisms. Here, we present an example of the evolution of insecticide resistance arising from transposable element–mediated disruption of a dominant insecticide-susceptible allele, resulting in expression of a recessive resistance allele and potent resistance. Our findings demonstrate how the adaptive potential of transposable elements can be revealed by environmental and genetic perturbation and that this can have profound and unexpected impacts on resistance. They also illustrate how combinations of mutations that individually confer no fitness benefit can interact to provide strong context-dependent fitness benefits.

The evolution of insecticide resistance represents a serious threat to the sustainable control of insect crop pests and disease vectors (1). In addition to its applied importance, the study of resistance also offers insights into a range of fundamental evolutionary questions concerning adaptation to environmental change, sources of adaptive variation, and the genetic architecture of novel traits (2).

Research into the proximate molecular mechanisms conferring reduced sensitivity to insecticides in a range of insect species has shown that resistance most commonly arises via two main mechanisms termed “metabolic resistance” and “target-site resistance.” Metabolic resistance results from increased activity or production of metabolic enzymes that detoxify or sequester the insecticide (3). In contrast, “target-site” resistance, which we consider in this study, results from structural changes (mutations) in the gene encoding the target protein, reducing its sensitivity to the toxic effect of the insecticide (4).

One of the first examples of target-site resistance in insects was the discovery of the molecular basis of knockdown resistance (kdr) to pyrethroid insecticides, a major class of neurotoxic insecticides that have been used extensively to control a wide range of agricultural and human health pests (5). Mutations in the protein target of this insecticide class, the voltage-gated sodium channel (VGSC), were shown to lead to reduced sensitivity of the insect nervous system to these compounds. The first, and most common, mutation identified in pyrethroid-resistant insect strains, referred to as “kdr,” arises from a single-point mutation in the S6 segment of domain II of the VGSC gene, resulting in a leucine to phenylalanine (L1014F) amino acid substitution (5, 6). Subsequently, secondary mutations giving enhanced (super kdr, “skdr”) resistance, such as the M918T substitution within the IIS4-S5 linker of the VGSC, were described (reviewed in ref. 7).

The green peach aphid *Myzus persicae* is a globally distributed, highly damaging crop pest (8). The economic importance of this species is increased by its extremely broad host range of more than 400 plant species, including many important crops, and its efficiency as a vector of more than 100 plant viruses (8). The intensive and widespread use of insecticides against this species has resulted in the evolution of resistance to many of the compounds used for control (9). This includes the pyrethroid insecticides where resistance has been linked to kdr/skdr mutations in the VGSC. Of these, the kdr L1014F and skdr M918T alterations are the most common, with M918T only ever found in combination with L1014F (i.e., on the same allele) (10, 11). More recently, an alternative substitution, M918L, at the skdr locus has been described and has been identified in the absence of L1014F (12, 13). The kdr and skdr mutations are inherited as recessive traits in a range of insect species, with little or no resistance phenotype observed in heterozygotes (5). Intriguingly, this does not appear to hold true in *M. persicae*, where aphid clones carrying L1014F, M918T, and M918L in the heterozygote state have been reported to display a resistant phenotype to a range of pyrethroids (11, 12), suggesting that kdr/skdr may be dominant in this species. Alternatively, the atypical mode of inheritance of kdr/skdr in *M. persicae* could be explained by interactions with other genetic elements that modify the phenotypic expression of these resistance alleles. In this regard, while knowledge of the individual genes and mutations that confer insecticide resistance has advanced dramatically over the last three decades, our understanding of the extent to which genetic variants interact to modify resistance phenotypes remains surprisingly poor.

In the current study, we investigated whether genetic factors distinct from known kdr/skr mutations modify the pyrethroid-resistance phenotype in *M. persicae*. We uncover an example of resistance arising from a mutation that silences a dominant insecticide susceptibility allele, resulting in a normally recessive resistance allele being phenotypically expressed.

## Results

### Atypical Pyrethroid Sensitivity Phenotype/Genotype Relationships Are Observed in Certain Clones of *M. persicae*.

Insecticide bioassays were used in combination with molecular genotyping to explore the relationship between kdr/skdr genotype and sensitivity to the pyrethroid bifenthrin in several clones of *M. persicae*. Three categories of insecticide sensitivity were observed, comprising 1) “susceptible” with a resistance ratio (RR) not greater than 3, as displayed by the reference susceptible clone 1X and clone 4H; 2) “resistant” with RR ∼1,500, as exhibited by clones 62H2 and 88H2; and 3) “highly resistant” with RR >10,000, as shown by clone 92H6 (Table 1). DNA genotyping of these clones revealed that, as expected, clone 1X was wild type at the kdr/skdr loci (susceptible genotype), consistent with the susceptibility of this clone (Table 1). Clone 92H6 was homozygous for both L1014F and M918T, explaining its extremely high resistance (Table 1). Unexpectedly, however, clones 4H, 62H2, and 88H2 were all heterozygous for both mutations (Table 1). As described in the introduction, kdr/skdr has been shown to be recessive in a range of insect species (5), and the susceptibility of clone 4H to bifenthrin is consistent with this. In contrast, the marked resistance of clones 62H2 and 88H suggests that either kdr/skdr is not inherited as a recessive trait in *M. persicae*, which is unlikely given the phenotype of 4H, or alternatively that additional genetic mechanisms exist in these clones, which confer resistance to bifenthrin or modify the phenotypic expression of kdr/sdr.

**Table 1.** Sensitivity of clones of *M. persicae* to the pyrethroid insecticide bifenthrin in full dose–response bioassays

Clone	LC_50_ (µg ⋅ mL^−1^)	CI 95%		Slope	RR	χ 2 (df)	*P* value	kdr genotype	skdr genotype
1X	1.07	0.56	1.67	1.65 ± 0.31		—	—	S/S	S/S
4H	3.19	1.83	4.95	1.13 ± 0.15	2.98	19.48 (2)	<0.01	S/R	S/R
62H2	1,563	682	6,056	0.97 ± 0.13	1,460	239 (2)	<0.01	S/R	S/R
88H2	1,606	1,107	2,325	1.12 ± 0.19	1,501	230 (2)	<0.01	S/R	S/R
92H6	10,830	3,961	434,640	0.71 ± 0.24	>10,000	204 (2)	<0.01	R/R	R/R

LC50: lethal concentration that is expected to cause 50% mortality; CI 95%: CI limits at 95%; RR: resistance ratio (calculated as the ratio between the LC50 of each clone and LC50 of the susceptible clone 1X). χ2 values (χ 2), degrees of freedom (df) and *P* values from likelihood ratio tests of equality of probit regressions of clone 1X with all other clones are detailed (null hypothesis is the slopes and intercepts of the probit regressions compared are the same). The genotype of clones for kdr and skdr are also shown (S: susceptible, R: resistant).

### Heterozygous Clones Differing in Bifenthrin Sensitivity Show Equivalent Expression of Detoxification Genes.

**Fig. 1.**
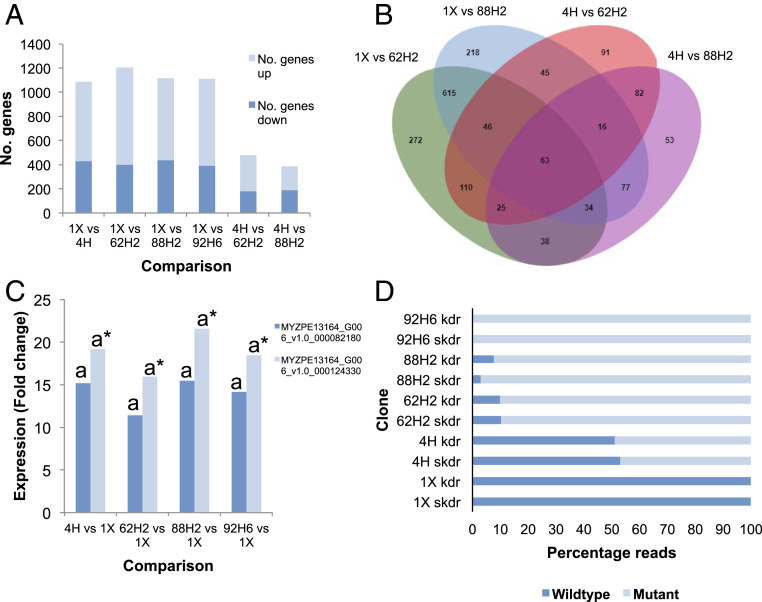
Clones of *M. persicae* that are heterozygous for the kdr + skdr allele but differ in sensitivity to bifenthrin show equivalent expression of genes encoding key detoxification enzymes but marked differences in the expression of the kdr + skdr and wild-type alleles. (*A*) Number of genes significantly differentially expressed in comparisons of RNA-seq data obtained from different *M. persicae* clones (*n* = 4). (*B*) Venn diagram displaying differentially expressed genes shared in different comparisons of bifenthrin-susceptible *M. persicae* clones (1X and 4H) with resistant (88H2 and 62H2) clones. (*C*) Fold change in expression of E4/FE4-like esterase genes (MYZPE13164_G006_v1.0_000082180 and MYZPE13164_G006_v1.0_000124330) in *M. persicae* clone comparisons (*n* = 4). Significant differences (*P* < 0.05) in expression for each gene between clones is denoted using letters above bars as determined by EdgeR (55) (Dataset S1), letters without asterisks designate significance of expression levels of MYZPE13164_G006_v1.0_000082180, and letters with asterisks designate significance of expression levels of MYZPE13164_G006_v1.0_000124330. (*D*) Expression of alleles of the VGSC with or without the kdr and skdr mutations as assessed by the percentage of RNA-seq reads with or without these mutations mapping at the kdr and skdr loci.

The marked difference in sensitivity to bifenthrin of heterozygous, resistant clones 62H2 and 88H2 compared to heterozygous, susceptible clone 4H could be explained by the presence of novel mutations in the VGSC in the former clones. Alternatively, the observed difference in sensitivity could arise from changes in the expression of metabolic enzymes that sequester, detoxify, or enhance the excretion of bifenthrin. To discriminate between these possibilities, we performed replicated RNA sequencing (RNA-seq) of all clones tested in the insecticide bioassays described above. RNA-seq data were mapped to the reference genome of the *M. persicae* G006 clone (14), and differentially expressed (DE) genes were called between the reference susceptible clone 1X and each of the other clones and between 4H and 62H2 and 88H2, as these three clones are all heterozygous for kdr + skdr but exhibit marked differences in sensitivity to bifenthrin (Table 1). Between 388 and 1,203 genes were identified as DE across these comparisons using a corrected false discovery rate cutoff of 0.05 and fold-change cutoff >2 (Fig. 1*A* and Dataset S1). Curation of genes that were DE in comparisons of both of the resistant clones 62H2 and 88H2 with both susceptible clones 1X and 4H resulted in a list of 63 DE genes, of which just 23 were consistently up-regulated and 13 down-regulated in both resistant clones (Fig. 1*B* and *SI Appendix*, Table S1). However, based on their biological function, none of these DE genes are strong candidates to explain the resistance of 62H2 and 88H2 to bifenthrin (*SI Appendix*, Table S1). We therefore used a candidate gene approach, using RNA-seq data, to examine the expression of genes encoding the carboxylesterases E4/FE4 among the clones, as these are the only metabolic enzymes that have been previously implicated in the resistance of *M. persicae* to pyrethroids (15). While *E4*/*FE4* were significantly overexpressed (11- to 22-fold) in all the clones analyzed relative to the susceptible reference clone 1X, no significant difference was observed in the level of E4/FE4 expression between 4H and either 62H2 or 88H2 (Fig. 1*C* and Dataset S1). Taken together, these results suggest that the resistance of clones 62H2 and 88H2 relative to 4H does not result from the enhanced expression of genes involved in insecticide detoxification.

### Bifenthrin-Resistant Clones Exhibit Monoallelic Expression of the kdr + skdr Resistance Allele.

To investigate if the pyrethroid resistance of clones 62H2 and 88H2 results from novel mutations in the VGSC, we mapped RNA-seq data to the two mature messenger RNA (mRNA) reference sequences encoding this protein (16). Compared to the reference sequence, no nonsynonymous mutations were observed in the RNA-seq reads of either clone, except at the position of the kdr (encoding L1014 in the wild type) and skdr (encoding M918 in the wild type) mutations. Thus, resistance to bifenthrin in these clones does not result from alternative skdr/kdr-type amino acid replacements. Surprisingly, however, marked differences in the expression of the wild-type and kdr + skdr allele were observed in both clones, with 90 to 97% of the RNA-seq reads mapping to the two mutation sites containing the kdr/skdr mutations (Fig. 1*D* and *SI Appendix*, Table S2). In contrast, when RNA-seq data of heterozygous, susceptible clone 4H was mapped to the VGSC, the expression of the mutant and wild-type allele was ∼50:50, as expected given the heterozygous genotype of this clone (Fig. 1*D* and *SI Appendix*, Table S2). To confirm the marked expression bias of the mutant and wild-type alleles observed in 62H2 and 88H2, fresh RNA was extracted from these clones and the region of the VGSC encompassing the two mutation sites was PCR amplified and cloned in *Escherichia coli*, with 30 of the resulting bacterial colonies derived from each clone sequenced. All the sequences returned from 62H2 and 88H2 carried the kdr + skdr allele, with no sequences representing the wild-type allele recovered (*SI Appendix*, Fig. S1), confirming the monoallelic expression of the pyrethroid-resistance allele of the VGSC in these clones.

### Transposon-Mediated Insertional Mutagenesis Silences Expression of the Dominant but Bifenthrin-Susceptible Allele in Resistant Clones.

**Fig. 2.**
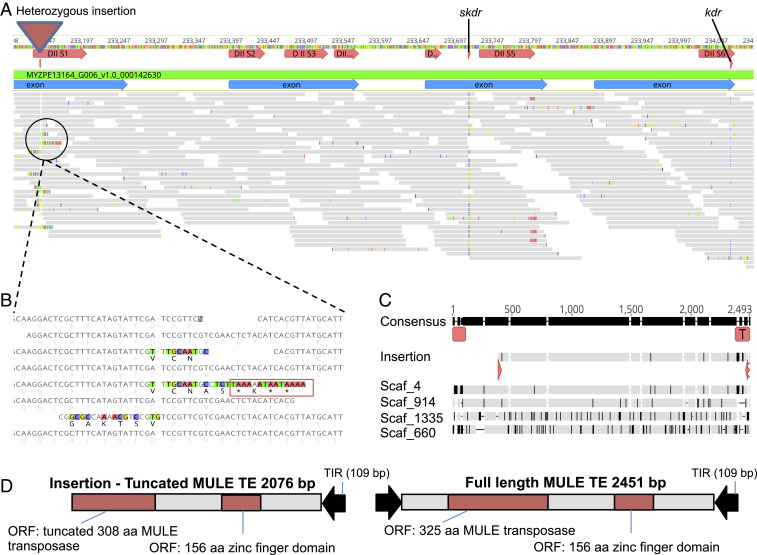
A heterozygous MULE insertion is observed in the coding sequence of the VGSC subunit 1 gene in clones 88H2 and 62H2 that introduces a premature termination codon. (*A*) DNA-seq reads mapped to the region of the VGSC gene encoding domains IIS1 to IIS6. (*B*) Close up of soft-clipped reads that diverge in sequence at the start of the region of VGSC encoding the IIS1 transmembrane helix domain that identify a heterozygous insertion that results in the introduction of several premature termination codons. (*C*) Alignment of the insertion against four sequences (with 96.6 to 99.9% sequence identity to the query sequence) obtained by BLAST searches against the *M. persicae* G006 reference genome. The gray regions indicate similarity between sequences, and black regions indicate sequence differences. Indels are indicated by gaps in the sequences. The 109-bp imperfect terminal inverted repeats that flank both ends of the returned sequences are indicated using red boxes on the consensus sequence. (*D*) Features of the truncated and full-length versions of the MULE transposon.

The marked reduction in expression of the wild-type allele of the VGSC subunit 1 gene may result from alterations in its regulatory regions or from nonfunctionalizing mutations in its coding sequence. To discriminate between these possibilities, the genomes of all study clones were resequenced and reads mapped to the scaffold on which the VGSC genes are located. Alignments were examined across the VGSC subunit 1 gene and ∼10-kb flanking regions to identify genetic variation (i.e., single nucleotide polymorphisms (SNPs), insertions and deletions (indels), etc.) that distinguish 62H2 and 88H2 from the other sequenced clones. Special attention was given to genetic variation present in 62H2 and 88H2 in the heterozygous form, as this would be consistent with the expression of just one allele of the VGSC in these clones. A single heterozygous SNP was observed in the 10-kb region upstream of the VGSC subunit 1 gene (1,891 base pairs [bp] upstream of the start codon), 6 heterozygous SNPs in the downstream region, 16 heterozygous SNPs and a single heterozygous insertion in intronic sequence, and a single heterozygous insertion in the coding sequence that distinguish 62H2 and 88H2 from the other sequenced aphid clones (*SI Appendix*, Table S3). Of these mutations, the position of the insertion (position 2,360 bp relative to the start codon of the mature mRNA) at the start of the region of the VGSC encoding the DIIS1 transmembrane helix, just 346 and 634 bp upstream of the position of the skdr and kdr mutation sites, respectively (Fig. 2*A*), made it the most promising candidate for a potential role in monoallelic expression of the kdr + skdr allele in 62H2 and 88H2. The characteristic soft-clipped reads (partially mapped reads that contain a contiguous region of sequence at their 5′ or 3′ end that does not match the reference) at position 2,360 bp that identify the insertion did not overlap when aligned (Fig. 2*B*). We therefore used iterative mapping of short reads to extend the left and right divergent sequences in silico to characterize the size and nature of the insertion at this position. Alignment of the extended sequences revealed that the insertion is 2,076 bp, and its size, sequence, and position were subsequently confirmed using PCR and Sanger sequencing. Interrogation of the final curated sequence (GenBank ID MW353161) against the National Center for Biotechnology Information (NCBI) non-redundant (nr) and Protein families (Pfam) databases revealed regions with homology to a Mutator-like element (MULE) transposase, suggesting the insertion was a DNA transposable element (TE) (Fig. 2*D*). BLAST searches of the full-length insertion against the reference genome of *M. persicae* clone G006 returned four sequences with 96.6 to 99.9% sequence identity to the query sequence, further suggesting its status as repetitive DNA. Alignment of these sequences revealed features characteristic of DNA transposons, such as 109-bp (imperfect) terminal inverted repeats flanking both ends of the sequence (Fig. 2 *C* and *D*). Finally, phylogenetic analysis of the insertion (using the alignment from ref. 17) placed the element within a clade containing other insect MULE elements, confirming its status as a DNA transposon belonging to the MULE superfamily (Dataset S2).

Examination of the MULE insertion site in the VGSC and the insertion sites of other copies in the *M. persicae* genome did not reveal any obvious sequence characteristics that might facilitate homing of this element or allow prediction of where it might insert. This is consistent with previous studies that have investigated the insertion specificity of MULE elements, which revealed an insertion preference for low copy number sequences but only weak preference for a specific target sequence consensus (17, 18).

Importantly, alignment of the MULE element identified from clones 62H2 and 88H2 with those obtained from the reference genome of G006 revealed that the sequence from the two bifenthrin-resistant clones is truncated at the 5′ end (Fig. 2*C*). In the context of its insertion in the VGSC, this results in the disruption of the reading frame from amino acid position 787 of the VGSC subunit 1, introducing multiple premature stop codons from position 792 (Fig. 2*B*). Thus, transcripts of the VGSC containing the MULE insertion would be expected to be eliminated by nonsense-mediated mRNA decay (NMD), the surveillance pathway in eukaryotes that targets mRNAs harboring premature stop codons for degradation in order to reduce errors in gene expression (19). Consequently, this would be predicted to result in effective down-regulation of the expression of the allele of the VGSC carrying the MULE insertion. VGSCs are multidomain proteins, consisting of four nonidentical domains (DI to DIV), with each domain comprising six transmembrane segments (S1 to S6) containing a voltage sensor (S1 to S4) and a membrane-spanning region (S5 to S6) (*SI Appendix*, Fig. S2). The MULE insertion occurs at the start of the region of the VGSC subunit 1 gene encoding the DIIS1 transmembrane region. Thus, the presence of premature stop codons in resulting transcripts that were not eliminated by NMD would result in their translation into a severely truncated protein that would lack critical domains that are essential for sodium channel function (16) (*SI Appendix*, Fig. S2**)**.

**Fig. 3.**
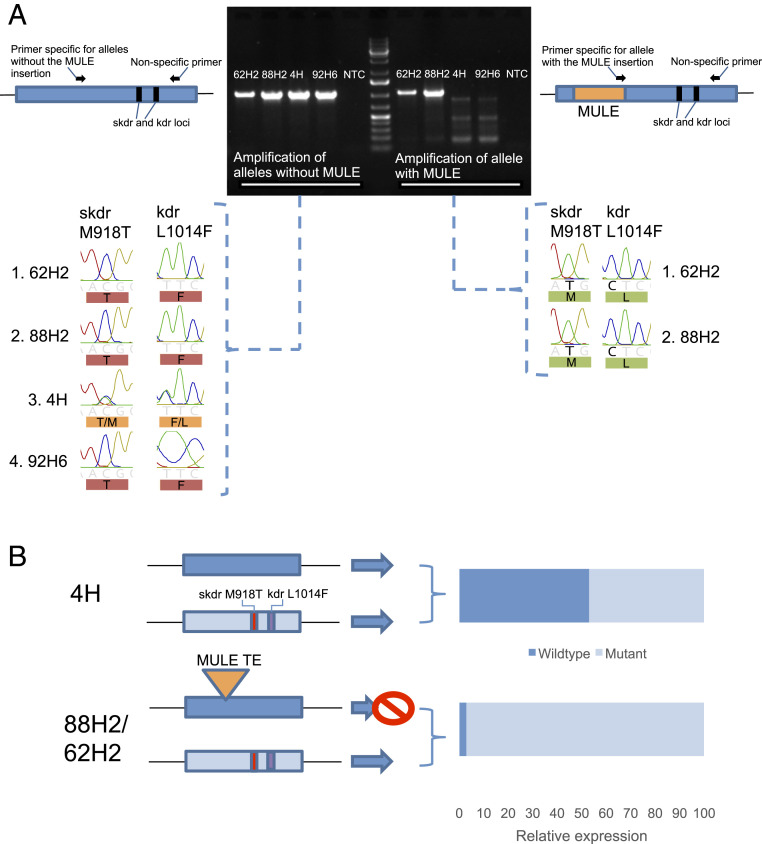
The MULE insertion down-regulates the expression of the bifenthrin-susceptible allele of the VGSC. (*A*) Results of PCR amplification and sequencing of alleles of the VGSC with or without the MULE insertion. Gel shows the results of PCR amplification from DNA of different aphid clones using primers specific for alleles of the VGSC with or without the MULE insertion, as illustrated in accompanying schematic. NTC: no template control. DNA size marker is the GeneRuler 1 kb plus DNA ladder (Thermo Fisher). Chromatograms show the genotype at the skdr and kdr loci obtained for each PCR product following sequencing. (*B*) Schematic of the impact of the MULE insertion on expression of the allele of the VGSC without the kdr + skdr mutations in clones 62H2 and 88H2 compared to clone 4H, which is heterozygous for kdr + skdr but lacks the MULE insertion. Expression level was inferred from RNA-seq data (*SI Appendix*, Table S2).

To examine whether the MULE element occurs on the “pyrethroid-susceptible” allele (i.e., the allele without the kdr + skdr mutations) or the “pyrethroid-resistant” allele (i.e., the allele carrying the kdr + skdr mutations), we designed primers that are specific for the allele with or without the MULE insertion (and that encompass the kdr and skdr sites) (Fig. 3*A* and *SI Appendix*, Table S4). PCR using the primers specific for the allele without the MULE insertion produced a band using DNA from all study clones as expected (Fig. 3*A*). In contrast, PCR using primers specific for the MULE insertion produced a band of the correct size only from DNA extracted from the 62H2 and 88H2 clones, confirming the exclusive insertion of the MULE element in these clones (Fig. 3*A*). Sequencing of the PCR products obtained using primers specific for the allele lacking the MULE insertion returned sequences from 62H2 and 88H2 with both the skdr (M918T) and kdr (L1014F) mutations (i.e., the pyrethroid-resistance allele) (Fig. 3*A*). Sequencing of the PCR products obtained using primers specific for the allele containing the MULE insertion returned sequences from these clones encoding the wild-type residues at the kdr and skdr positions (i.e., the insecticide-sensitive allele) (Fig. 3*A*). These findings demonstrate that the MULE insertion is present on the allele of the VGSC lacking kdr + skdr, and the premature stop codons it introduces are the molecular explanation for the down-regulation of this allele in both 62H2 and 88H2. Thus, despite the fact that these clones are heterozygous for skdr (M918T) and kdr (L1014F), they express only functional VGSC isoforms that carry the resistance mutations, making them insensitive to the effects of the pyrethroid bifenthrin (Fig. 3*B*). Our finding that just 3 to 10% of the RNA-seq reads mapping to the sites of the kdr/skdr mutations encode wild-type residues is consistent with this. The low levels of transcripts representing the allele without kdr + skdr could be explained by the fact that NMD does not always result in 100% silencing (20). Alternatively, the fact that NMD is a translation-coupled mechanism (19) and thus takes place in the cytoplasm means that RNA-seq reads derived from mRNA extracted from the nucleus of cells would not yet have been degraded by this mechanism.

To investigate the prevalence of the novel resistance mechanism in field populations of *M. persicae*, we developed a simple PCR diagnostic to detect the presence of the allele of the VGSC carrying the MULE insertion (*SI Appendix*, Fig. S3) and used it to screen 148 aphid clones collected from peach in Italy in 2019 (*SI Appendix*, Table S5 and Fig. S4). As peach is the primary host of *M. persicae*, aphid clones derived from this host plant are expected to be holocyclic (i.e., capable of reproducing sexually). The TE insertion was observed in 8/148 clones (5.4%), of which two were heterozygous for classical kdr + skdr (M918T) (*SI Appendix*, Table S5). Interestingly, the remaining six clones with the MULE insertion were heterozygous for an alternative form of skdr (M918L), raising the possibility that monoallelic expression of this allele also confers resistance in *M. persicae* (*SI Appendix*, Table S5). PCR and sequencing confirmed that the MULE insertion occurs on the “pyrethroid-susceptible” allele (i.e., lacking skdr [M918L]) in these clones. Thus, we would expect this to lead to the degradation of transcripts encoding the pyrethroid-susceptible allele and monoallelic expression of the allele carrying skdr (M918L).

### Monoallelic Expression of the VGSC Subunit 1 Reduces Total Expression Levels and Results in a Temperature-Dependent Fitness Cost.

**Fig. 4.**
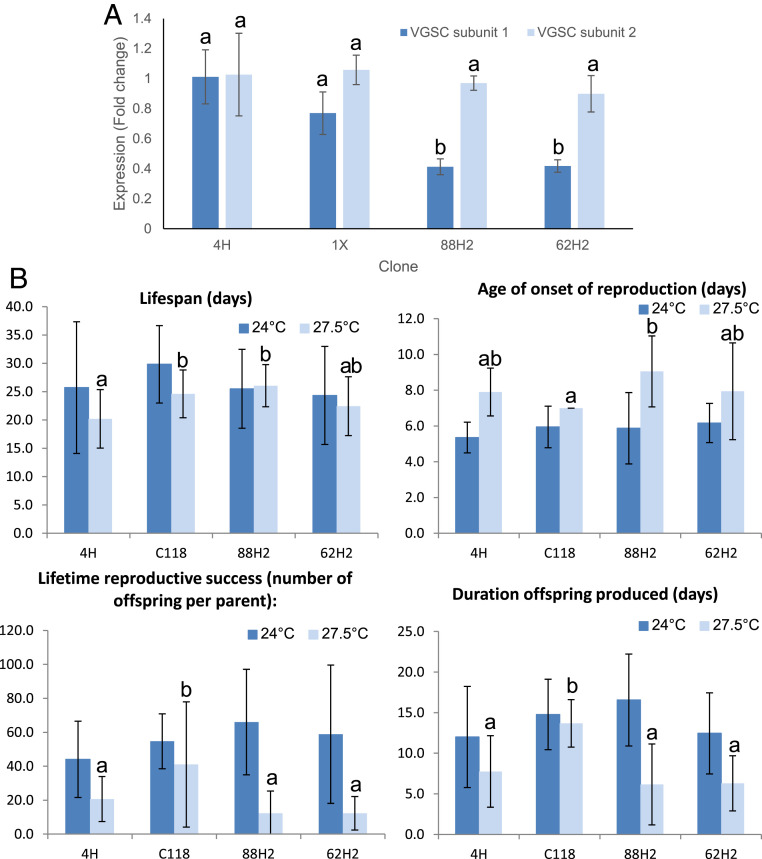
Monoallelic expression of the VGSC subunit 1 reduces total expression levels and results in a temperature-dependent reproductive fitness cost. (*A*) qPCR analysis of the expression of the VGSC subunit 1 and 2 genes in *M. persicae* clones with (62H2 and 88H2) and without (1X and 4H) the MULE insertion. Expression is shown as fold change relative to clone 4H. The error bars show 95% CI (*n* = 4). Significant differences (*P* < 0.01) in expression between clones is denoted using letters above bars as determined by one-way ANOVA with post hoc testing (Tukey Honestly Significant Difference [HSD]). (*B*) Measurement of reproductive fitness and lifespan of *M. persicae* clones that are all heterozygous for kdr + skdr but carry this resistance allele in combination with an allele of the VGSC with (62H2 and 88H2) or without (C118 and 4H) the MULE insertion at 24 and 27.5 °C. The error bars display SD (*n* = 18). Significant differences (*P* < 0.01) in fitness measurement between clones at 27.5 °C is denoted using letters above bars as determined by one-way ANOVA with post hoc testing (Tukey HSD). No significant differences between clones for any fitness measurement were observed at 24 °C.

To examine the consequences of monoallelic expression of the VGSC subunit 1 gene on the total expression levels of this gene, we compared the expression of the mRNAs encoding the first and second subunits of the VGSC, which in aphids are coded for by different genes (16), in the study clones using qPCR. While no difference in the expression of the gene encoding the second VGSC subunit was observed between clones, the expression of the gene encoding the first subunit was ∼50% lower in heterozygous-resistant clones 88H2 and 62H2 compared to either the homozygous-susceptible clone 1X, or the heterozygous-susceptible clone 4H (Fig. 4*A*). Thus, the monoallelic expression of the allele carrying kdr + skdr in 88H2 and 62H2 results in an ∼50% reduction in the expression of the VGSC subunit 1 gene. The VGSC is an essential channel that is required for initiation and propagation of action potentials in the nervous system. Thus, it is unsurprising that mutations in VGSC genes that alter the quantity of viable transcript have been implicated in a variety of neural disorders in humans (21). Furthermore, mutations that reduce the expression of the VGSC in *Drosophila* are associated with increased temperature-sensitive paralysis and reduced fitness (22). For example, flies carrying the *maleless* mutant allele (*mle*^*napts*^) show reduced expression of the VGSC and exhibit temperature-sensitive developmental lethality, decreased fecundity, and increased neurodegeneration (22). Given these findings, it might be expected that the ∼50% reduction observed in the expression of the VGSC subunit 1 gene in *M. persicae* clones carrying the MULE insertion would result in similar fitness costs. To test this, we compared the lifespan and lifetime reproductive success of clones 88H2 and 62H2 to that of clones 4H and a further clone, C118. Like clone 4H, C118 expresses both kdr + skdr and wild-type alleles and is susceptible to bifenthrin (lethal concentration 50% [LC_50_] of 1.36 µg ⋅ mL^−1^, 95% CI 1.09, 2.05). Under laboratory conditions at 24 °C, no significant difference was observed in lifespan, lifetime reproductive rate, or age of reproductive onset (Fig. 4*B*). However, at 27.5 °C, the lifetime reproductive success of both clone 88H2 and 62H2 was significantly (*P* < 0.0001 in both cases) lower (73 and 71% lower, respectively) than that of clone C118 and 48 and 43% lower than clone 4H, although comparisons with the latter did not reach statistical significance at *P* < 0.05 (Fig. 4*B*). Thus, the reduced expression of the VGSC in clones 88H2 and 62H2 appears to be associated with a temperature-dependent reduction in reproductive fitness.

## Discussion

Our data uncover a mechanism of insecticide resistance resulting from the interaction of a recessive resistance allele with genetic variation created by a TE. These findings provide fundamental insights into the role of TEs as a source of evolutionary potential that can be unlocked by environmental and genetic perturbation, with applied implications for the control of highly damaging insect pests. We discuss these topics below.

TEs are a major component of insect genomes; however, the extent of their role in insect evolution remains unclear (23). As potent mutagens, most TE insertions have nearly neutral or negative effects on host fitness (24). However, in the context of the evolution of insecticide resistance, TE insertions can lead to resistance by up-regulating genes encoding insecticide detoxifying enzymes or by disrupting insecticide receptors leading to insecticide insensitivity (25–27). Here, we demonstrate a more complex case of the involvement of TEs in insecticide resistance. In isolation, the MULE TE insertion we identify in the VGSC subunit 1 gene confers no resistance to pyrethroids. Indeed, it introduces a premature termination codon into an allele of an essential gene that dramatically reduces its expression and results in a severely truncated protein lacking critical domains essential for function (*SI Appendix*, Fig. S2). The phenotype associated with loss-of-function mutations of the VGSC gene in *Drosophila* (where it is named *para*) is unconditional recessive lethality (28, 29). Particularly relevant to our study, the *para*^*hd5*^ allele carries a P element transposon insertion in its coding sequence that results in the death of *para*^*hd5*^*/para*^*hd5*^ early-stage larvae (29, 30). Furthermore, RNA interference knockdown of the VGSC subunit 1 gene in *M. persicae* by oral feeding of double-stranded RNA was shown to result in a 2.5-fold reduction in expression and caused up to 65% mortality in third instar nymphs (31). Thus, in the homozygous state, the MULE insertion in the VGSC would be expected to be lethal. Furthermore, we show that clones carrying the mutation in the heterozygous state exhibit a marked reduction in lifetime reproductive success at higher temperatures. This temperature-dependent fitness cost is consistent with reports of temperature-sensitive paralysis and reduced fitness of *Drosophila* strains with mutations that reduce expression of the VGSC (22). However, the fact that fitness costs were not apparent at 24 °C, together with the field-derived nature of these clones, demonstrates that clones carrying the mutation in the heterozygous form can successfully persist outside of a laboratory environment.

Thus, in the context of resistance to insecticides, in the heterozygous state, the MULE insertion represents a context-dependent deleterious genetic variant that, in isolation, provides no direct phenotypic advantage in the presence of insecticides. However, we show that in combination with an allele carrying kdr + skdr, the MULE insertion transforms the phenotypic expression of a normally recessive resistance allele into one that confers strong insensitivity to pyrethroids. This results from the introduction of premature termination codons into the VGSC by the MULE insertion, effectively disrupting the expression of the susceptible allele, leading to the monoallelic expression of the allele carrying the resistance mutations (Fig. 3*B*). Consequently, only VGSCs with kdr + skdr, which are insensitive to the effects of pyrethroid insecticides, are produced. These findings provide a compelling example of how TEs, which are nearly neutral or deleterious under most conditions, can provide a source of rapid adaptive potential under conditions of environmental change, such as exposure to insecticides, or in combination with a particular genetic background. In this regard, analyses of *Drosophila melanogaster* population genomic data have shown that a large fraction of TE insertions in this species are not fixed, with the majority segregating among populations at low frequency (32), constituting a large cache of standing genetic variation for natural selection to act upon. Furthermore, in organisms that reproduce clonally, the activity of TEs may provide an even more important means of maintaining a source of genetic variation in large populations for selection to act upon.

The findings of our study display parallels with previous research on recessive pesticide-resistance mutations in other insects, while also exhibiting some important differences. Firstly, in insect and mite species with a haplodiploid sex-determination system, or where one sex is heterogametic, the hemizygous sex may be resistant because the phenotype of a recessive resistance allele is not masked by its dominant susceptible counterpart (33–35). Secondly, loss-of-function mutations in insecticide target sites have been shown to confer recessive resistance to insecticides previously. For example, numerous mutations in the nicotinic acetylcholine receptor alpha six subunit have been reported that lead to the production of truncated nonfunctional protein and resistance to spinosyns—the pesticides that target this receptor (36–38). Similarly, a range of loss-of-function mutations in cadherin-encoding genes, including TE insertions, confer recessive resistance to *Bacillus thuringiensis* toxins, with resistant individuals carrying two alleles with the same or different mutations (27, 39–41). However, in both these cases, the nature and diversity of mutations observed reflects the fact that these insecticide target sites are not essential for viability, thus any loss-of-function mutation will confer resistance without compromising viability (37, 41). In contrast, the VGSC is essential for life (29), and we would predict strong functional constraints on the nature and location of resistance-associated mutations in this receptor (42). Work over the last three decades has validated this prediction, with the same or very similar point mutations identified at a limited number of positions in the VGSC gene of a variety of different insect species (5). The same is true for other genes encoding essential proteins targeted by insecticides (4, 43). Thus, our findings provide a rare example that nonsense mutations in essential genes can, in the right genetic background and under conditions of high selection, provide a mechanism for resistance.

The evolution of this mechanism of insecticide resistance is intriguing given that the same end result (resistance) can be achieved simply by inheriting two copies of the allele bearing kdr + skdr. Indeed, as exemplified in this study by clone 92H6, clones of *M. persicae* that are homozygous for these mutations exhibit very strong (>10,000-fold) resistance to pyrethroids. The higher levels of resistance observed in kdr + skdr homozygous clones, compared to clones with a single kdr + skdr allele in combination with an allele with the MULE insertion, is unclear. However, it may reflect a fitness cost associated with the MULE insertion, which makes them more susceptible to the secondary mode of action of pyrethroid insecticides in inducing oxidative damage, rather than their primary neurotoxicity (44). Regardless, given the strong resistance of kdr + skdr homozygotes, a key question is how a mutation has persisted that is predicted to be strongly deleterious in the homozygous form and which only provides fitness benefits in the presence of the kdr + skdr allele? A possible explanation is provided by the mode of reproduction of *M. persicae*. The typical lifecycle of this species comprises a yearly cycle of sexual reproduction (holocycly) alternating with parthenogenetic (asexual) reproduction (anholocycly). However, in some countries, *M. persicae* reproduces continuously by parthenogenesis or has both holocyclic and anholocyclic life cycles depending on genotype and environment (8). Thus, while in obligate sexually reproducing insects, alleles will be regularly shuffled by segregation and recombination; in insects reproducing asexually, such as anholocyclic *M. persicae*, alleles (and combinations of alleles) can become essentially locked in the same clone. This situation can favor the persistence of alleles that are (in isolation) deleterious and the spread of allele combinations that have fitness benefits. Furthermore, the primarily asexual mode of reproduction in *M. persicae* means that the chance of resistance arising from crosses of individuals that are heterozygous for recessive resistance mechanisms is considerably lower than in exclusively sexually reproducing species. However, de novo mutations do occur in asexually reproducing aphid lineages (45), and MULE elements are known for their very high rates of transposition (17). Thus, the MULE insertion could have arisen in a lineage of *M. persicae* carrying kdr + skdr during the asexual component of its life cycle. In this context, the MULE insertion would have conferred an immediate fitness benefit in the presence of pyrethroid insecticides, and the probability of this mutation surviving loss via stochastic processes or purifying selection would have been significantly increased.

However, the fact that we observe the identical MULE insertion in two genetically distinct clones of *M. persicae* provides clear evidence that this allele has spread between genotypes by sexual reproduction at some point. In this context, it is notable that crosses of clones with an allele containing the MULE insertion and an allele carrying the kdr + skdr mutation with clones without the MULE insertion that carry kdr + skdr in the heterozygous or homozygous form are predicted to produce 50 and 100% of bifenthrin-resistant progeny (*SI Appendix*, Fig. S5). This is equivalent to the percentage of resistant progeny predicted to be produced by crosses of clones that are homozygous for kdr + skdr with clones that are kdr + skdr homozygotes or heterozygotes and exceeds the percentage of resistant progeny produced by crosses of kdr + skdr heterozygotes, where just 25% of the progeny would be expected to be resistant to pyrethroids (*SI Appendix*, Fig. S5). Furthermore, while crosses of clones that both carry the MULE insertion in combination with kdr + skdr would be expected to result in 25% nonviable progeny as a result of them carrying two copies of the allele with the MULE insertion, the remaining 75% of progeny would be resistant to bifenthrin (*SI Appendix*, Fig. S5). To summarize, in populations where kdr + skdr is prevalent, the effect of the MULE insertion on the dominance of these resistance alleles would favor its spread in the presence of pyrethroid insecticides even in holocyclic populations and despite the fact it is almost certainly homozygous lethal. Consistent with this prediction, we observe the MULE insertion in combination with kdr + skdr in recent field-collected *M. persicae* from peach. Aphids collected from this host are expected to be holocyclic, providing evidence that the MULE insertion can persist in holocyclic populations in combination with kdr + skdr under pyrethroid selection. In contrast, in populations without kdr + skdr, the VGSC allele with the MULE insertion would provide no fitness benefits and would not be predicted to increase in frequency.

From an applied perspective, our results illustrate the critical role genetic background can have on the phenotypic expression of known resistance genes, raising implications for insecticide-resistance monitoring and management programs. The majority of resistance mutations in insecticide target sites are intrinsically recessive (46). Thus, it is commonly assumed that pest populations that exhibit a high frequency of individuals carrying such mutations in the heterozygous form are largely susceptible to insecticides. Our data provide further evidence of the importance of supplementing the use of molecular assays to diagnose resistance in field populations with regular measurement of phenotypes using insecticide bioassays. Given the large numbers of active TEs present in the genomes of many insects coupled with large population sizes and strong selection mediated by insecticide treatment, it is likely that many more cases of the involvement of TEs in resistance phenotypes will be uncovered as further genomes are screened.

In conclusion, we present an example of the evolution of insecticide resistance resulting from the interaction of distinct allelic variants that in isolation confer no fitness advantages. Our findings serve as a demonstration of how effectively natural selection tests combinations of random mutations arising in populations and allows those that confer an enhanced ability to survive and reproduce to proliferate. In this regard, our study also illustrates how this process may be facilitated in organisms that reproduce asexually by allowing adaptive combinations of alleles to become locked in clonal genomes.

## Materials and Methods

### Aphid Clones.

The reference susceptible strain 1X was collected in Tuscany, Italy, in 1995 from peach. Clones 4H and 62H2 were both collected in peach orchards located in Ravenna, Italy, in 1998 and 1999, respectively. Clone 88H2 was collected from peach in Dossobuono, Italy, in 2003. Clone C118 was collected in the United Kingdom from Brussels Sprouts in 1997. Clone 92H6 was selected from a resistant population collected after neonicotinoid control failures in peach orchards in 2010 in Cesena. All clones were reared asexually on pea seedlings (cultivar Meraviglia d’Italia) in controlled environmental conditions (21 ± 0.5 °C with a 16:8 h light:dark photoperiod) as reported previously (47).

### Insecticide Bioassays.

The sensitivity of *M. persicae* clones to the pyrethroid bifenthrin (Brigata Flo; SIPCAM) was assessed by full dose–response insecticide dip-test bioassays as previously described (13). Samples were maintained at 21 ± 0.5 °C with a 16:8 h photoperiod. Mortality was assessed 24 h after pyrethroid application. Probit analysis of concentration-mortality relationships, calculation of 95% fiducial limits, and likelihood ratio tests for equality of probit regressions were performed using the POLO-Plus software (LE-ORA Software).

### *kdr* and *skdr* Genotyping.

Genomic DNA was extracted using the GenElute Mammalian Genomic DNA Miniprep Kit (Sigma-Aldrich) according to the manufacturer’s instructions. DNA fragments 600 bp in size, encompassing the skdr and kdr sites, were PCR amplified with primers *kdr*-F1 and *kdr*-R4 (*SI Appendix*, Table S4). PCR reactions (25 μL) contained 12.5 μL of DreamTaq Green PCR Master Mix (Thermo Scientific), 0.4 μM of each primer, and 1 μL of genomic DNA. Thermocycling conditions comprised 2 min at 94 °C, followed by 30 cycles of 94 °C for 30 s, 52 °C for 30 s, and 72 °C for 1 min, followed by a final incubation at 72 °C for 10 min. PCR products were purified using the GenElute PCR Clean-Up Kit (Sigma-Aldrich), quantified using a Qubit Fluorimeter 2.0 (Quant-iT ds DNA HS Assay kit; Invitrogen) and direct sequenced on both strands using the same primers. Chromatograms were aligned and inspected using Geneious Software version 10 (Biomatters).

### RNA-Seq Analyses.

Aphid clones were age synchronized under identical environmental conditions and four biological replicates each comprising 10 apterous female aphids 14 d in age pooled for RNA extraction using the ISOLATE II RNA Mini Kit (Bioline). RNA was used as a template for the generation of barcoded libraries (TrueSeq RNA library preparation, Illumina), which were then sequenced to >10M paired-end (PE) reads per replicate on an Illumina HiSeq2500 flowcell using a 150-bp PE read metric at the Earlham Institute. All sequence data has been deposited with the NCBI Short Read archive as BioProject PRJNA574571 and run accession numbers: SRR13327277 (4H), SRR13327274 (92H6), SRR10199549 (1X), SRR13327276 (62H2), and SRR13327275 (88H2). The quality of the reads obtained was assessed using FASTQC version 0.11.5 (48) and adaptor sequences and low-quality base calls removed using TrimGalore 0.4.5 (49). Clean reads were aligned to the G006v2 genome assembly using HISAT2 version 2.1.0 (50) and gene expression estimated using the HTSeq count tool implemented in the HTSeq package (51). EdgeR version 3.9 (52) was used to identify significantly DE genes using a corrected *P* value threshold of *P* < 0.05 and a fold change >2.

To examine the relative expression levels of alleles of the VGSC with and without the kdr + skdr mutations and to investigate if the pyrethroid resistance of the 62H2 or 88H clones results from novel mutations in the VGSC, RNA-seq data were mapped to the mature mRNA reference sequences encoding this protein (GenBank accessions: FN601405 and FN601406) using the map to reference function in Geneious version 10 (Biomatters). To verify the patterns of expression of VGSC alleles observed in RNA-seq analyses, total RNA was isolated from fresh aphids as described above, and first-strand complementary DNA (cDNA) synthesized from 1 μg of the total RNA using RevertAid H Minus First Strand cDNA Synthesis Kit (Thermo Scientific) according to the manufacturer’s protocol. PCR was used to amplify an 825-bp region of the VGSC subunit 1 gene that encompasses the kdr and skdr sites using primers MpSK-F25 and MpSK-R21 (*SI Appendix*, Table S4). PCR reactions (25 μL) contained 12.5 μL of DreamTaq Green PCR Master Mix (Thermo Scientific), 0.4 μM of each primer, and 2 μL of cDNA. Temperature cycling conditions comprised 2 min at 94 °C, followed by 30 cycles of 94 °C for 30 s, 58 °C for 30 s, and 72 °C for 2 min, with a final elongation at 72 °C for 10 min. PCR products obtained were purified with the GenElute PCR Clean-Up Kit (Sigma-Aldrich), quantified with a Qubit Fluorimeter 2.0 (Quant-iT ds DNA HS Assay kit; Invitrogen), and either direct sequenced on both strands using the same primers or cloned prior to sequencing. For the latter, purified PCR products were ligated into the pJET 1.2 vector of the GeneJET PCR cloning kit (Thermo Scientific) and transformed into XL-1 blue cells (Agilent). Plasmids containing an insert of the expected size were purified using the GeneJET miniprep kit (Thermo Scientific), following the manufacturer’s protocol and sequenced as above. Chromatograms were aligned and inspected using Geneious Software version 10 (Biomatters).

### Resequencing of *M. persicae* Genomes and Identification of Variants in the VGSC Gene.

DNA was extracted from pools of 10 to 20 aphids of each clone using the E.Z.N.A. Insect DNA Kit (Omega Bio-tek) and used to construct PCR-free libraries. Libraries were sequenced on a NovaSeq6000 using a 150-bp PE read metric to an average coverage of 40×. FastQC was used to check the quality of the raw reads obtained (48), and reads were trimmed using TrimGalore (49). Sequence data are available at NCBI under the BioProject ID: PRJNA574571 and run accession numbers: SRR13326477 (1X), SRR13326474 (4H), SRR13326473 (62H2), SRR13326471 (88H2), and SRR13326470 (92H6). Trimmed reads were mapped to the scaffold containing the VGSC genes (AphidBase Clone G006, scaffold 5, gene id: MYZPE13164_G006_v1.0_000142630.1), using the map to reference function in Geneious version 10 (Biomatters). Alignments were manually inspected to identify variation in the VGSC gene and 10-kb flanking regions that distinguish 62H2 and 88H2 from the other sequenced clones.

### Bioinformatic, Molecular, and Phylogenetic Analyses of the MULE TE Insertion.

The MULE insertion in the VGSC subunit 1 gene was first characterized using iterative mapping of short reads to extend the left and right divergent sequences identified in the short DNA-seq reads mapping over the insertion site in silico. This was conducted using the “map to reference” function of Geneious version 10 using the low-sensitivity (highest stringency) option, with the extended left and right regions aligned each mapping round until they overlapped. The final sequence obtained was confirmed by conventional PCR and sequencing using the primers MULE_F3 and MULE_R (*SI Appendix*, Table S4) and the conditions described above but using a 3-min extension time in PCR.

The open reading frame of the identified *M. persicae* MULE element was identified and aligned to the MULE superfamily-wide amino acid alignment of Dupeyron et al. (17) using MUSCLE version 3.8 (53). The resultant alignment was used to estimate a maximum likelihood phylogeny using IQTree version 2.0 (54) with 1,000 bootstrap replicates.

PCR and sequencing was used to examine whether the MULE element occurs on the allele without kdr + skdr mutations (i.e., the pyrethroid-susceptible allele) or the allele carrying the kdr + skdr mutations (i.e., the pyrethroid-resistant allele), using primers that are specific for the alleles with (MT_F1) or without (WT_F1) the MULE insertion (and that encompass the kdr and skdr sites) in combination with the generic “reverse” primer kdrgDNA_IntR2 (*SI Appendix*, Table S4). Thus, in aphid clones with the MULE insertion, products of the correct size (1,105 bp for WT_F1+ kdrgDNA_IntR2 and 1,106 bp for MT_F1+ kdrgDNA_IntR2) are produced in PCR using both primer combinations. However, in aphid clones without the insertion, products of the correct size are only produced using the primer combination specific for alleles without the MULE insertion (WT_F1+ kdrgDNA_IntR2). PCR conditions were as described above, with products purified and sequenced on both strands using the same primers and following the methods detailed above.

### Development of a PCR Diagnostic for the MULE Insertion and Screening of Field-Collected *M. persicae.*

A PCR diagnostic for the MULE insertion in the VGSC subunit 1 gene was developed that employs primers that are specific for the allele with (MT_F2) or without (WT_F1) the MULE insertion (and that encompass the kdr and skdr sites) in combination with the generic “reverse” primers R1 and R2 (*SI Appendix*, Table S4). In the presence of the VGSC allele with the MULE insertion the primer combinations MT_F2 and R1 generate a 326-bp product and the primer combination MT_F2 and R2 a product of 276 bp. In the presence of the allele without the MULE insertion, the primer combinations WT_F1 and R1 generate a 327-bp product. The PCR diagnostic was used to screen for the allele of the VGSC carrying the MULE insertion in 148 clones collected from peach (*Prunus persica*) in Italy in 2019 (*SI Appendix*, Table S5 and Fig. S4). PCR reactions (25 μL) contained 12.5 μL of DreamTaq Green PCR Master Mix (Thermo Scientific), 0.4 μM of each primer, and 1 μL of DNA. Temperature cycling conditions comprised 2 min at 95 °C, followed by 35 cycles of 95 °C for 30 s, 59 °C for 30 s, and 72 °C for 40 s, with a final elongation at 72 °C for 5 min. Following amplification, PCR products were separated by electrophoresis on a 2% agarose gel in Tris borate ethylenediaminetetraacetic acid (EDTA) buffer.

### QPCR.

QPCR was used to compare the expression of the genes encoding the two subunits of the VGSC between clones of *M. persicae* using the conditions described previously (55) and the primers shown in *SI Appendix*, Table S4. Data were analyzed according to the ΔΔC_T_ method (56), using the geometric mean of two housekeeping genes (actin and *para*) for normalization according to the strategy described previously (57).

### Fitness Assays.

To assess reproductive fitness of *M. persicae* clones, single aphids of each clone (18 replicates per clone) were allowed to mature and produce nymphs in individual containers, supported on a single leaf of *Brassica rapa*, at 24 ± 1 or 27.5 ± 1 °C and a photoperiod of 16:8. Containers were monitored regularly to ensure the continued quality of the food source, with leaves replaced as required. Days until first offspring were produced, total offspring, number of reproductive days, and the lifespan of each individual was recorded.

### Quantification and Statistical Analysis.

All statistical analyses were performed in GraphPad Prism 7 (GraphPad Software). Significant differences in expression or copy number in qPCR experiments and in fitness assays were determined using one-way ANOVA with post hoc Tukey honestly significant difference (HSD). Statistical details of experiments (value of *n*, precision measures, and definitions of significance) are provided in figure legends.

## Supplementary Material

Supplementary File

Supplementary File

Supplementary File

## Data Availability

The DNA-seq and RNA-seq data generated in this study is available at NCBI under the Bio Project ID: PRJNA574571 (58). The truncated and full-length MULE sequences are available at NCBI under the accession nos. MW353161 (59) and MW353162 (60), respectively. All other study data are included in the article and/or supporting information.
