# Supplementary material for: Transposon-mediated insertional mutagenesis unmasks recessive insecticide resistance in the aphid Myzus persicae
Source: Proc Natl Acad Sci U S A. 2021 May 31;118(23):e2100559118. doi: 10.1073/pnas.2100559118 (PMC8201860; doi:10.1073/pnas.2100559118)
Supplement: Supplementary File [file pnas.2100559118.sapp.pdf]

PNAS
www.pnas.org

Supplementary Information for

Transposon-mediated insertional mutagenesis unmasks recessive insecticide resistance in the aphid, *Myzus persicae*

Michela Panini[1], Olga Chiesa[1], Bartlomiej J Troczka[2], Mark Mallott[2], Gian Carlo Manicardi[3], Stefano Cassanelli[3], Filippo Cominelli[1], Alex Hayward[2], Emanuele Mazzoni[1*], Chris Bass[2*]

[1]Department of Sustainable Crop Production, Section Sustainable Crop and Food Protection, Università Cattolica del Sacro Cuore, Piacenza, Italy

[2]College of Life and Environmental Sciences, Biosciences, University of Exeter, Penryn Campus, Penryn, Cornwall, UK

[3]Dipartimento di Scienze della Vita, Università di Modena e Reggio Emilia, Via Amendola 2, 42122, Modena, Italy

*Emanuele Mazzoni

*Chris Bass

Email: c.bass@exeter.ac.uk emanuele.mazzoni@unicatt.it

This PDF file includes:

Figures S1 to S5

Tables S1 to S5

Legends for Datasets S1 to S2

Other supplementary materials for this manuscript include the following:

Datasets S1 to S2

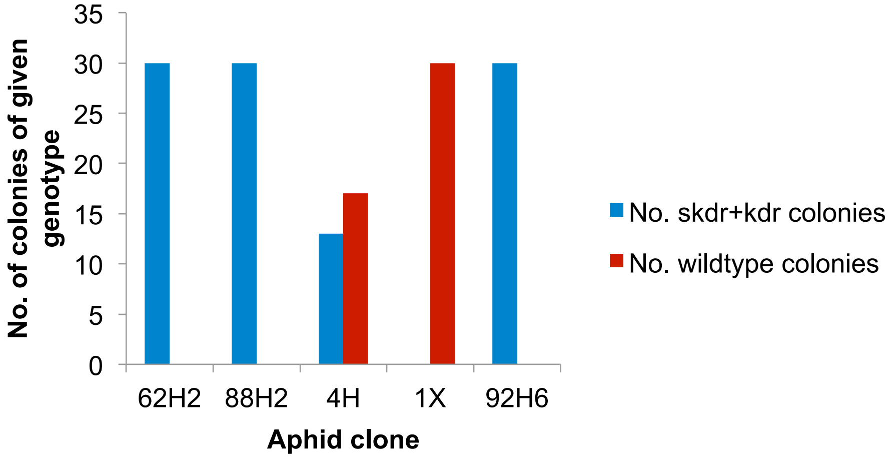

Fig. S1. Relative expression of the wild-type and kdr+skdr alleles of the VGSC subunit 1 gene in bifenthrin resistant (62H2, 88H2 and 92H6) and susceptible (1X and 4H) clones of *Myzus persicae* as determined by cloning and sequencing. The number of sequenced colonies containing the kdr+skdr allele or wild-type allele following PCR amplification and cloning of an amplicon encompassing the kdr and skdr mutation sites is shown for each clone.

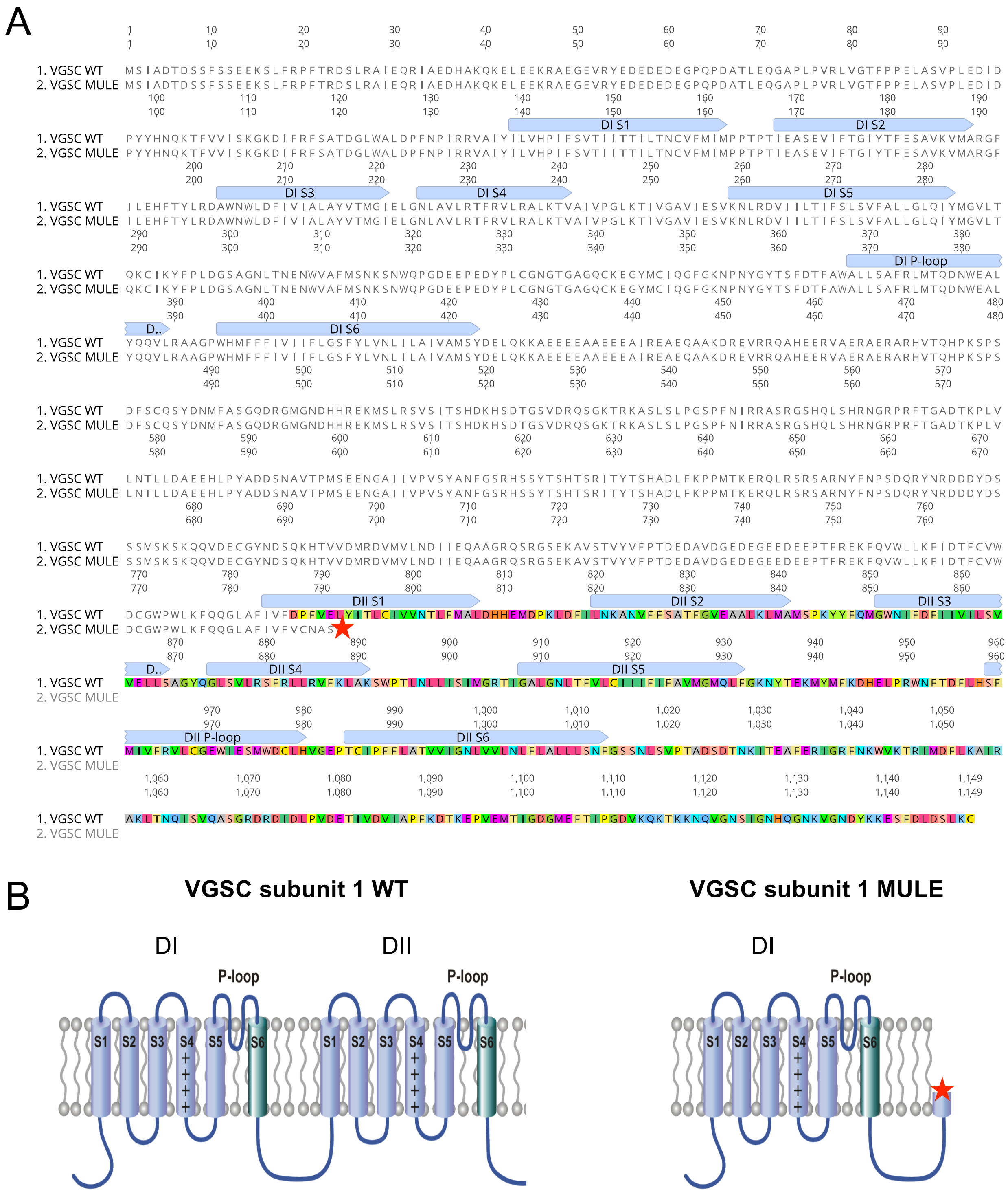

Fig. S2. Impact of the MULE insertion on the VGSC subunit 1 protein. (A) Alignment of the primary translated open reading fames of the VGSC subunit 1 gene, with (VGSC MULE) and without (VGSC WT) the MULE insertion. Differences between the two sequences are highlighted. The first premature stop codon introduced by the MULE insertion is indicated by a red star. The amino acid sequence of important functional domains such as the six transmembrane segments (S1–S6) of each domain (DI and DII) and the P-loops, are annotated above the sequence. (B) Schematic of the VGSC subunit 1 protein produced from each of the sequences shown in panel A. The wildtype protein comprises two non-identical domains (DI, and DII), with each domain comprising six transmembrane segments (S1–S6) containing a voltage sensor (S1–S4) and a membrane-spanning pore region (S5–S6). The introduction of premature stop codons by the MULE insertion (indicated by a red star) would result in a truncated protein lacking most of domain II.

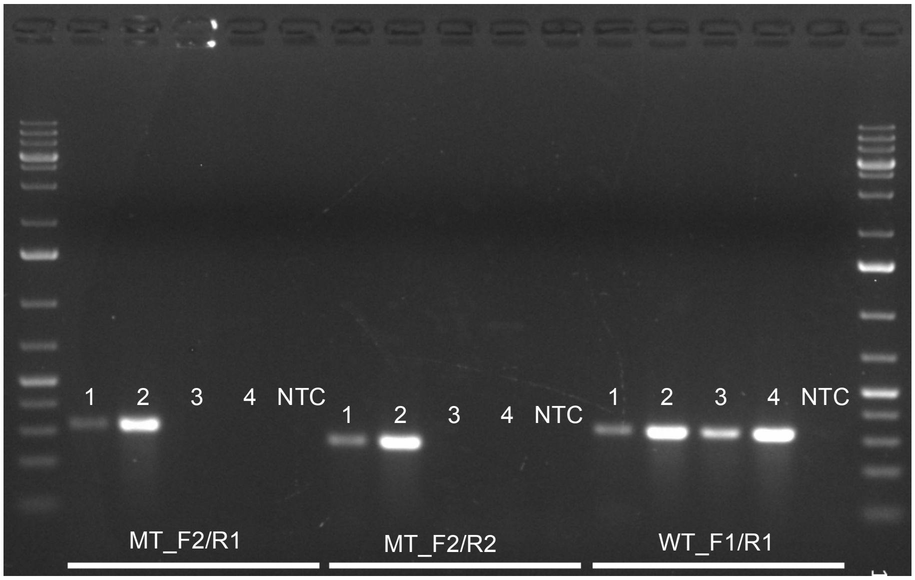

Fig. S3. Development of a PCR diagnostic for the MULE insertion in the VGSC subunit 1 gene. Gel shows the results of PCR amplification from DNA of *Myzus persicae* clone 62H2 (lane 1), 88H2 (lane 2), 4H (lane 3) and 92H6 (lane 4) using primers specific for alleles of the VGSC with, or without, the MULE insertion. NTC: no template control. DNA size marker is the GeneRuler 1kb Plus DNA ladder (ThermoFisher). In the presence of the VGSC allele with the MULE insertion the primer combinations MT_F2 and R1 generate a 326 bp product and the primer combination MT_F2 and R2 a product of 276 bp. In the presence of the allele without the MULE insertion the primer combinations WT_F1 and R1 generate a 327 bp product.

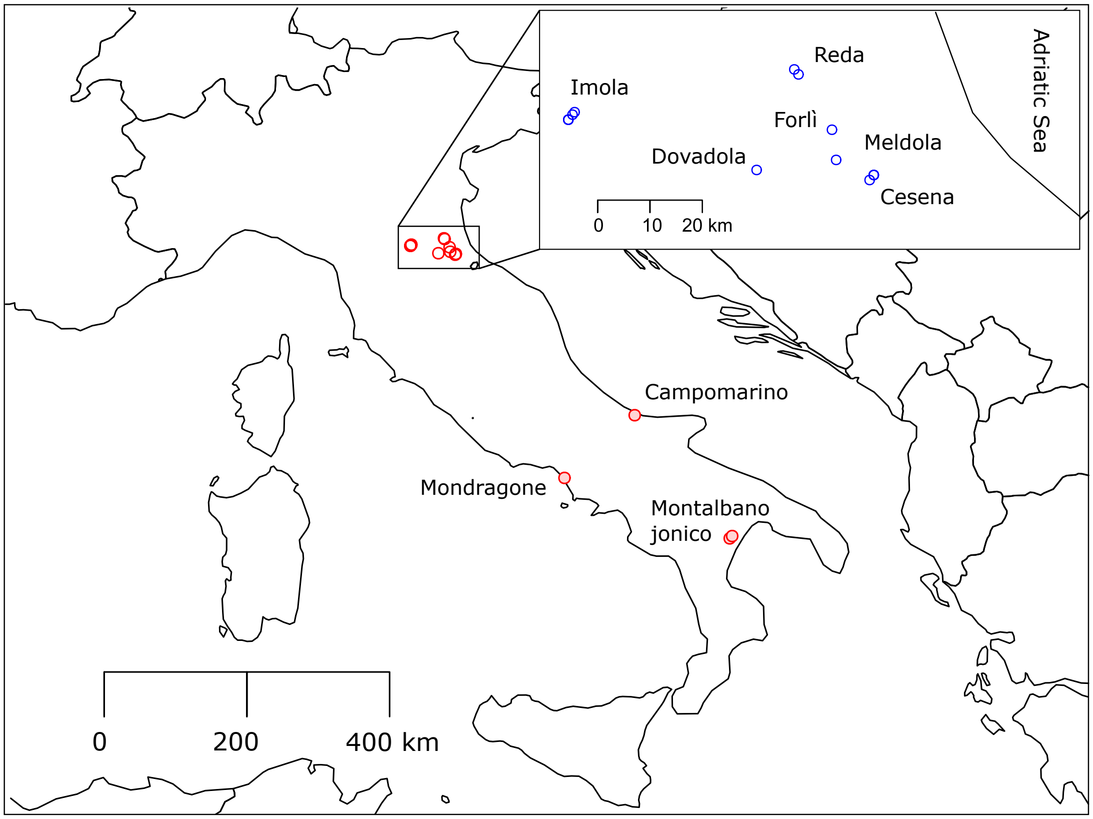

Fig. S4. Geographic origin of *Myzus persicae* samples collected in Italy for molecular genotyping.

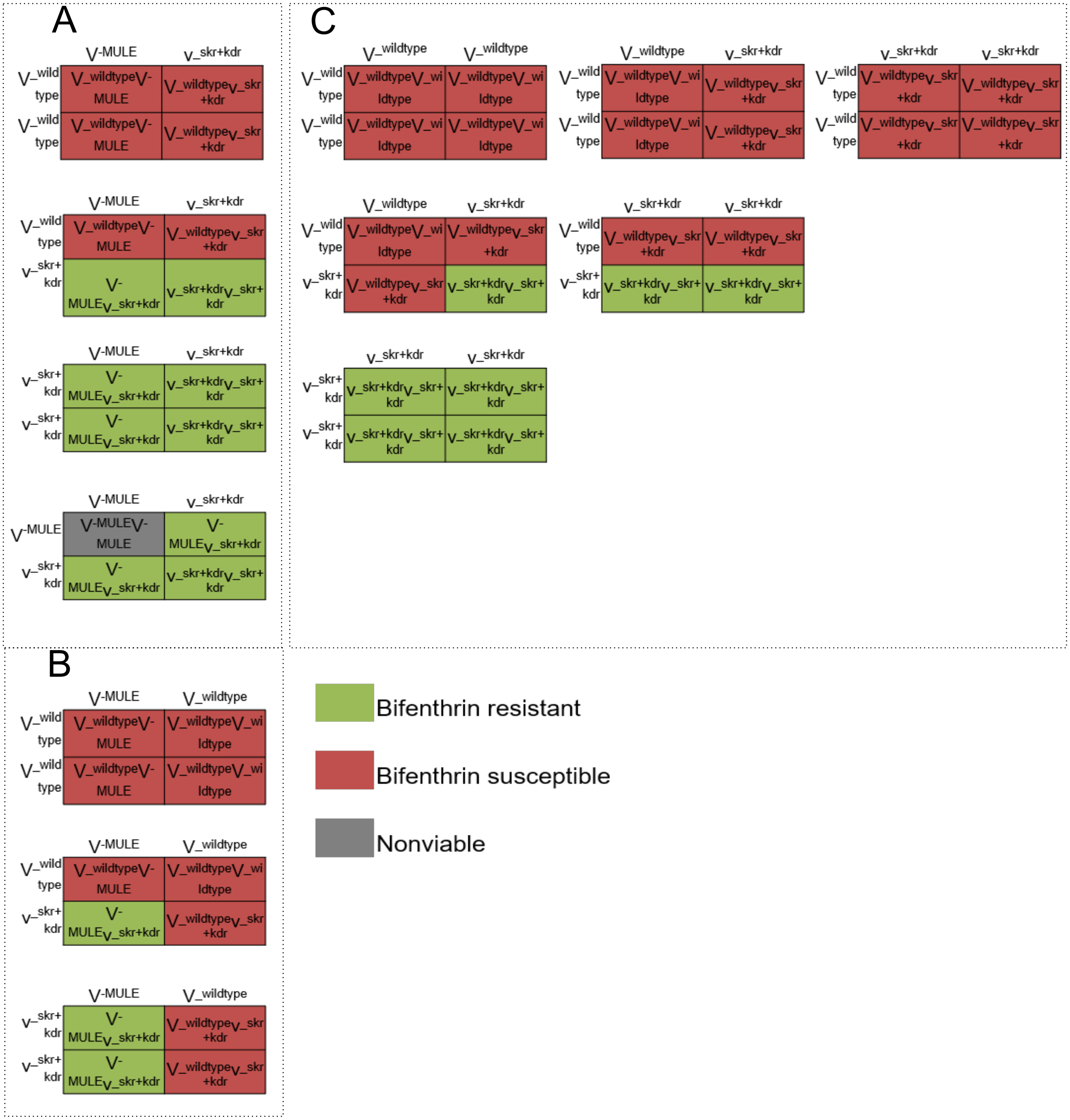

Fig. S5. Expected bifenthrin sensitivity genotypes and phenotypes in the offspring of crosses of *Myzus persicae* clones with and without the MULE insertion. (A) Percentage genotypes/phenotypes produced from crosses of *M. persicae* clones carrying an allele of the VGSC with the MULE insertion in combination with an allele with the skdr+kdr mutations (V_MULE /V_skdr+kdr), with clones without the MULE insertion and of varying skdr+kdr genotype. (B) Percentage genotypes/phenotypes produced from crosses of clones carrying an allele of the VGSC with the MULE insertion in combination with a wildtype allele (i.e. lacking the skdr+kdr mutations) (V_MULE /V_wildtype), with clones without the MULE insertion but varying in skdr+kdr genotype. (C) Percentage genotypes/phenotypes produced from crosses of clones varying in skdr+kdr genotype (provided for comparison).

6

Table S1. List of genes consistently upregulated or downreguated (false discovery rate (FDR) < 0.05 and fold change (FC) > 2) in both of the bifenthrin resistant *Myzus persicae* clones 62H2 and 88H2 compared to both of the bifenthrin susceptible clones 1X and 4H as determined by RNAseq analyses.

	1X_vs_62H2_FC	1X_vs_62H2_FDR	1X_vs_88H2_FC	1X_vs_88H2_FDR	4H_vs_62H2_FC	4H_vs_62H2_FDR	4H_vs_88H2_FC	4H_vs_88H2_FDR	AHRD_description
MYZPE13164_G006_v1.0_000079110	1551.7	0.0000	2930.0	0.0000	6736.0	0.0000	12542.4	0.0000	Zinc finger BED domain-containing protein 1
MYZPE13164_G006_v1.0_000131780	699.5	0.0000	416.7	0.0000	844.7	0.0000	511.4	0.0000	Unknown protein
MYZPE13164_G006_v1.0_000120620	438.5	0.0000	340.3	0.0000	515.4	0.0000	403.3	0.0000	Unknown protein
MYZPE13164_G006_v1.0_000168180	199.3	0.0000	152.1	0.0000	79.4	0.0000	63.9	0.0000	Unknown protein
MYZPE13164_G006_v1.0_000196700	35.8	0.0000	27.4	0.0000	3.0	0.0001	2.3	0.0151	Tol2 transposase
MYZPE13164_G006_v1.0_000090880	27.9	0.0000	23.8	0.0000	14.1	0.0000	12.4	0.0000	Nuclease harbi1-like protein
MYZPE13164_G006_v1.0_000114030	9.4	0.0000	2.3	0.0141	12.2	0.0000	2.6	0.0051	Unknown protein
MYZPE13164_G006_v1.0_000050510	8.9	0.0000	9.4	0.0000	9.5	0.0000	10.1	0.0000	A disintegrin and metalloproteinase with thrombospondin motifs
MYZPE13164_G006_v1.0_000196350	8.0	0.0000	8.0	0.0000	7.1	0.0000	7.5	0.0000	Chorion peroxidase
MYZPE13164_G006_v1.0_000050500	7.8	0.0000	2.1	0.0199	17.0	0.0000	4.5	0.0000	Voltage-dependent T-type calcium channel subunit alpha-1G
MYZPE13164_G006_v1.0_000050470	7.1	0.0000	3.2	0.0000	8.2	0.0000	3.7	0.0000	Voltage-dependent T-type calcium channel subunit alpha-1H
MYZPE13164_G006_v1.0_000014700	6.6	0.0000	4.0	0.0000	5.3	0.0000	3.2	0.0000	Ubiquitin-like-specific protease 1
MYZPE13164_G006_v1.0_000019730	5.5	0.0000	3.0	0.0011	33.0	0.0000	17.8	0.0000	Retrovirus-related Pol polyprotein from transposon 17.6
MYZPE13164_G006_v1.0_000048450	5.2	0.0000	2.7	0.0161	4.3	0.0000	2.1	0.0434	Unknown protein
MYZPE13164_G006_v1.0_000054860	4.3	0.0000	6.9	0.0000	2.0	0.0110	3.2	0.0000	Unknown protein
MYZPE13164_G006_v1.0_000188970	4.1	0.0000	3.9	0.0000	2.1	0.0003	2.1	0.0054	UvrABC system protein B
MYZPE13164_G006_v1.0_000201890	3.8	0.0000	3.4	0.0000	2.9	0.0000	2.7	0.0000	Gamma-glutamyltranspeptidase 1
MYZPE13164_G006_v1.0_000057120	3.7	0.0000	3.4	0.0001	8.0	0.0000	7.5	0.0000	Tigger transposable element-derived protein 6-like protein
MYZPE13164_G006_v1.0_000175230	3.7	0.0000	2.1	0.0028	5.7	0.0000	3.3	0.0010	Unknown protein
MYZPE13164_G006_v1.0_000094570	3.3	0.0005	6.5	0.0000	5.2	0.0000	10.1	0.0000	Zinc finger mym-type protein 1-like protein

MYZPE13164_ G006_v1.0_000 166740	2.6	0.0000	7.3	0.0000	3.2	0.0000	9.1	0.0000	Kelch-like protein 2/3
MYZPE13164_ G006_v1.0_000 026680	2.3	0.0094	2.4	0.0131	8.5	0.0000	8.5	0.0000	ACYPI001252 protein
MYZPE13164_ G006_v1.0_000 033510	2.3	0.0000	2.3	0.0000	3.5	0.0000	3.7	0.0000	PR domain zinc finger protein 12
MYZPE13164_ G006_v1.0_000 165400	-2.2	0.0002	-2.3	0.0000	-2.3	0.0018	-2.4	0.0000	KRAB-A domain-containing protein 2
MYZPE13164_ G006_v1.0_000 102510	-2.2	0.0000	-2.2	0.0000	-2.0	0.0000	-2.0	0.0000	Armadillo repeat-containing protein 6
MYZPE13164_ G006_v1.0_000 178110	-2.4	0.0010	-3.4	0.0000	-2.1	0.0060	-3.1	0.0000	Kelch-like protein 17
MYZPE13164_ G006_v1.0_000 037230	-2.8	0.0000	-2.0	0.0001	-4.2	0.0000	-3.1	0.0000	Wd-repeat protein
MYZPE13164_ G006_v1.0_000 052540	-2.9	0.0000	-3.6	0.0000	-2.1	0.0062	-2.8	0.0004	Tol2 transposase
MYZPE13164_ G006_v1.0_000 171530	-3.7	0.0000	-2.8	0.0012	-3.0	0.0043	-2.3	0.0309	Unknown protein
MYZPE13164_ G006_v1.0_000 176310	-3.9	0.0000	-7.6	0.0000	-2.5	0.0056	-4.7	0.0000	Unknown protein
MYZPE13164_ G006_v1.0_000 158910	-4.2	0.0000	-4.0	0.0000	-2.5	0.0092	-2.3	0.0231	Unknown protein
MYZPE13164_ G006_v1.0_000 077370	-4.4	0.0000	-5.6	0.0000	-3.2	0.0000	-3.9	0.0000	SpeE2 protein
MYZPE13164_ G006_v1.0_000 164380	-10.6	0.0000	-3.0	0.0000	-8.6	0.0000	-2.4	0.0000	Mitochondrial ubiquitin ligase activator of nfkb 1-A
MYZPE13164_ G006_v1.0_000 197500	-30.5	0.0000	-4.2	0.0000	-24.2	0.0000	-3.2	0.0003	Coiled-coil domain-containing protein 65
MYZPE13164_ G006_v1.0_000 056580	-232.9	0.0000	-219.3	0.0000	-220.4	0.0000	-209.5	0.0000	Nuclease harbi1
MYZPE13164_ G006_v1.0_000 194870	-359.2	0.0000	-8.5	0.0000	-584.4	0.0000	-11.2	0.0000	Zinc finger MYM-type protein 1

Table S2. Relative expression of the wild-type and kdr+skdr alleles of the VGSC subunit 1 gene in bifenthrin resistant (62H2, 88H2 and 92H6) and susceptible (1X and 4H) clones of *Myzus persicae* as determined by RNAseq analysis. The percentage of RNAseq reads that carry the wild-type or mutant bases, that map over the position of the kdr (encoding amino acid position 1014) and skdr (encoding amino acid position 918) mutation sites, is shown.

Clone	Percentage reads M918	Percentage reads T918	Read coverage at M918T	Percentage reads L1014F	Percentage reads F1014	Read coverage at L1014F
1X	100	0	185	100	0	163
4H	53.1	46.9	209	51.1	48.9	368
62H2	10.3	89.7	155	9.8	90.2	205
88H2	2.9	97.1	138	7.6	92.4	197
92H6	0	100	254	0	100	278

Table S3. Genetic variation, present in the heterozygous state, across the gene encoding the first subunit of the VGSC and ~10kb flanking regions that distinguish the *Myzus persicae* clones 62H2 and 88H2 from clones 92H6, 1X, and 4H. The position of each variant on scaffold 5 of the G006v2 genome assembly is indicated.

Position on scaffold	Mutation	Gene region
1546869	T>C	Upstream region
1549308	T>C	Intronic
1552330	A>G	Intronic
1556085	A>C	Intronic
1558044	G>A	Intronic
1558201	A>G	Intronic
1558465	T>C	Intronic
1558473	C>A	Intronic
1559513	C>G	Intronic
1560744	A>G	Intronic
1561754	indel (2076 bp)	CDS
1563614	A>T	Intronic
1563650	A>T	Intronic
1563882	A>C	Intronic
1563923	A>G	Intronic
1564271	indel (10 bp)	Intronic
1565473	G>A	Intronic
1567091	C>T	Intronic
1569797	A>C	Downstream region
1570936	G>A	Downstream region
1571342	A>T	Downstream region
1573099	A>C	Downstream region
1573118	A>G	Downstream region
1575238	A>C	Downstream region

Table S4. Sequences of the oligonucleotide primers used in this study.

Name	Sequence (5'-3')	Use
kdr-F1	TCGTGGCCCACACTGAATCT	Genotyping for kdr and skdr using gDNA
kdr-R4	GTTCATGTAAGATACATGAATTC	Genotyping for kdr and skdr using gDNA
MpSK-F25	TGAAACTGATGGCGATGAGCCCTA	Genotyping for kdr and skdr using cDNA
MpSK-R21	TCCCGTCACCAATTGTCATCTCCA	Genotyping for kdr and skdr using cDNA
MULE_F3	ATA GTA TTC GTT TGC AAT GCC TC	Sequence characterisation of MULE insertion
MULE_R	TTC GTG ATG ATC GAG GGC CA	Sequence characterisation of MULE insertion
MT F1	AAA CGG CGG CGC CAA AAC G	Specific for the allele with the MULE insertion
MT F2	GGC GCC AAA ACG TCC GTG T	Specific for the allele with the MULE insertion
WT F1	CGC TTT CAT AGT ATT CGA TCC G	Specific for the allele without the MULE insertion
Kdr_gDNA_IntR2	TGA TAA TAG AAG ATG GTT GTA GGT TC	Generic reverse primer for use with MTF1/F2 and WT_F1
R1	TAG GGC TCA TCG CCA TCA GT	Generic reverse primer for use with MTF1/F2 and WT_F1
R2	ACT GCA ATC GTA TTC GGA ATT AG	Generic reverse primer for use with MTF1/F2 and WT_F1
NaChF1	TTCAAAGACCACGAGCTTCC	qPCR of the VGSC subunit 1 gene
NachR1	CGACGTGTAAACAGTCCCAC	qPCR of the VGSC subunit 1 gene
NaChF3	TTGGTCAACGCGTCTAGGAT	qPCR of the VGSC subunit 2 gene
NaChR3	ACTGAAGATCCACAAGCCGA	qPCR of the VGSC subunit 2 gene
Actin_F	GGTGTCTCACACACAGTGCC	qPCR of reference gene (actin)
Actin_R	CGGCGGTGGTGGTGAAGCTG	qPCR of reference gene (actin)
Para_F	GACCACGAGCTTCCCCGGTG	qPCR of reference gene (voltage-gated sodium channel)
Para_R	TGGTATACACGTTGGTTCTC	qPCR of reference gene (voltage-gated sodium channel)

Table S5. Results of screening 148 clones of *Myzus persicae* collected in Italy in 2019 from peach (*Prunus persica*) for the allele of the VGSC subunit 1 gene containing the MULE insertion. For all clones the corresponding kdr and skdr genotype is shown for each allele using the code S for the pyrethroid susceptible residue and R for the resistant residue.

Specimen ID	Sampling date	Location	TE insertion	kdr genotype	skdr genotype	918 codons
Mp-221.01	26/03/19	Imola (BO)	-	SR	RR	ACG / CTG
Mp-221.02	26/03/19	Imola (BO)	-	RR	RR	ACG / ACG
Mp-221.03	26/03/19	Imola (BO)	-	SR	RR	ACG / TTG
Mp-221.04	26/03/19	Imola (BO)	-	SS	RR	CTG / CTG
Mp-221.05	26/03/19	Imola (BO)	-	RR	RR	ACG / ACG
Mp-221.06	26/03/19	Imola (BO)	-	SR	SR	ATG / ACG
Mp-221.07	26/03/19	Imola (BO)	-	RR	RR	ACG / ACG
Mp-221.08	26/03/19	Imola (BO)	+	SS	SR	ATG / TTG
Mp-221.09	26/03/19	Imola (BO)	+	SS	SR	ATG / TTG
Mp-221.10	26/03/19	Imola (BO)	+	SS	SR	ATG / TTG
Mp-221.11	26/03/19	Imola (BO)	+	SS	SR	ATG / TTG
Mp-221.12	26/03/19	Imola (BO)	+	SR	SR	ACG / TTG
Mp-222.01	26/03/19	Imola (BO)	-	RR	RR	ACG / ACG
Mp-222.02	26/03/19	Imola (BO)	-	SR	SR	ATG / ACG
Mp-222.03	26/03/19	Imola (BO)	-	RR	RR	ACG / ACG
Mp-222.04	26/03/19	Imola (BO)	-	RR	RR	ACG / ACG
Mp-222.05	26/03/19	Imola (BO)	-	RR	RR	ACG / ACG
Mp-222.06	26/03/19	Imola (BO)	-	RR	RR	ACG / ACG
Mp-222.07	26/03/19	Imola (BO)	-	SR	RR	ACG / CTG
Mp-222.08	26/03/19	Imola (BO)	-	SR	RR	ACG / CTG
Mp-222.09	26/03/19	Imola (BO)	-	SR	RR	ACG / CTG
Mp-222.10	26/03/19	Imola (BO)	-	SR	RR	ACG / CTG
Mp-222.11	26/03/19	Imola (BO)	-	SR	SR	ATG / ACG
Mp-222.12	26/03/19	Imola (BO)	-	SR	SR	ATG / ACG
Mp-226.A1	09/05/19	Imola (BO)	-	SR	RR	ACG / CTG
Mp-226.A2	09/05/19	Imola (BO)	-	SR	RR	ACG / CTG
Mp-226.A3	09/05/19	Imola (BO)	-	SR	RR	ACG / CTG
Mp-226.A4	09/05/19	Imola (BO)	-	RR	RR	ACG / ACG
Mp-226.A5	09/05/19	Imola (BO)	-	SR	RR	ACG / CTG
Mp-226.B1	09/05/19	Imola (BO)	-	SR	RR	ACG / CTG
Mp-226.B2	09/05/19	Imola (BO)	-	SR	RR	ACG / CTG
Mp-226.B3	09/05/19	Imola (BO)	-	SR	RR	ACG / CTG
Mp-226.B4	09/05/19	Imola (BO)	-	SR	RR	ACG / CTG
Mp-226.B5	09/05/19	Imola (BO)	-	SR	RR	ACG / CTG
Mp-226.C1	09/05/19	Imola (BO)	-	SR	RR	ACG / CTG
Mp-226.C2	09/05/19	Imola (BO)	-	SR	RR	ACG / CTG
Mp-226.C3	09/05/19	Imola (BO)	-	SR	RR	ACG / CTG
Mp-226.C4	09/05/19	Imola (BO)	-	SR	RR	ACG / CTG
Mp-226.C5	09/05/19	Imola (BO)	-	SR	RR	ACG / CTG
Mp-226.D1	09/05/19	Imola (BO)	-	SR	RR	ACG / CTG
Mp-226.D2	09/05/19	Imola (BO)	-	SR	RR	ACG / CTG
Mp-226.D3	09/05/19	Imola (BO)	-	SR	RR	ACG / CTG
Mp-226.D4	09/05/19	Imola (BO)	-	SR	RR	ACG / CTG
Mp-226.D5	09/05/19	Imola (BO)	-	SR	RR	ACG / CTG
Mp-227.01	13/05/19	Montalbano Jonico (MT)	-	SR	RR	ACG / CTG
Mp-227.02	13/05/19	Montalbano Jonico (MT)	-	SR	RR	ACG / CTG
Mp-227.03	13/05/19	Montalbano Jonico (MT)	-	SR	SR	ATG / CTG
Mp-227.04	13/05/19	Montalbano Jonico (MT)	-	SR	RR	ACG / CTG
Mp-227.05	13/05/19	Montalbano Jonico (MT)	-	SR	RR	ACG / CTG
Mp-227.06	13/05/19	Montalbano	-	SR	RR	ACG / CTG

		Jonico (MT)				
Mp-227.07	13/05/19	Montalbano Jonico (MT)	-	SR	RR	ACG / TTG
Mp-227.08	13/05/19	Montalbano Jonico (MT)	-	SR	RR	ACG / TTG
Mp-227.09	13/05/19	Montalbano Jonico (MT)	-	SR	RR	ACG / TTG
Mp-227.10	13/05/19	Montalbano Jonico (MT)	-	SR	RR	ACG / CTG
Mp-227.11	13/05/19	Montalbano Jonico (MT)	-	SR	RR	ACG / CTG
Mp-227.12	13/05/19	Montalbano Jonico (MT)	-	SR	RR	ACG / TTG
Mp-228.01	13/05/19	Montalbano Jonico (MT)	-	SR	RR	ACG / CTG
Mp-228.02	13/05/19	Montalbano Jonico (MT)	-	SR	SS	ATG / ATG
Mp-228.03	13/05/19	Montalbano Jonico (MT)	-	RR	RR	ACG / ACG
Mp-228.04	13/05/19	Montalbano Jonico (MT)	-	RR	RR	ACG / ACG
Mp-228.05	13/05/19	Montalbano Jonico (MT)	-	SR	RR	ACG / CTG
Mp-228.06	13/05/19	Montalbano Jonico (MT)	-	SS	SS	ATG / ATG
Mp-228.07	13/05/19	Montalbano Jonico (MT)	-	RR	RR	ACG / ACG
Mp-228.08	13/05/19	Montalbano Jonico (MT)	-	SS	SR	ATG / CTG
Mp-228.09	13/05/19	Montalbano Jonico (MT)	-	SS	SR	ATG / CTG
Mp-228.10	13/05/19	Montalbano Jonico (MT)	-	SS	SS	ATG / ATG
Mp-228.11	13/05/19	Montalbano Jonico (MT)	-	SS	SR	ATG / CTG
Mp-228.12	13/05/19	Montalbano Jonico (MT)	-	RR	SS	ATG / ATG
Mp-229.01	16/05/19	Imola (BO)	-	SR	RR	ACG / CTG
Mp-229.02	16/05/19	Imola (BO)	-	SR	RR	ACG / CTG
Mp-229.03	16/05/19	Imola (BO)	-	SR	RR	ACG / CTG
Mp-229.04	16/05/19	Imola (BO)	-	RR	RR	ACG / ACG
Mp-229.05	16/05/19	Imola (BO)	-	RR	RR	ACG / ACG
Mp-229.06	16/05/19	Imola (BO)	-	SR	RR	ACG / TTG
Mp-229.07	16/05/19	Imola (BO)	-	RR	RR	ACG / ACG
Mp-229.08	16/05/19	Imola (BO)	-	SR	RR	ACG / CTG
Mp-229.09	16/05/19	Imola (BO)	-	RR	RR	ACG / ACG
Mp-229.10	16/05/19	Imola (BO)	-	RR	RR	ACG / ACG
Mp-229.11	16/05/19	Imola (BO)	-	SR	RR	ACG / CTG
Mp-229.12	16/05/19	Imola (BO)	-	RR	RR	ACG / ACG
Mp-230.01	16/05/19	Imola (BO)	-	SR	RR	ACG / CTG
Mp-230.02	16/05/19	Imola (BO)	-	RR	RR	ACG / ACG
Mp-230.03	16/05/19	Imola (BO)	-	SR	RR	ACG / CTG
Mp-230.04	16/05/19	Imola (BO)	-	SR	RR	ACG / CTG
Mp-230.05	16/05/19	Imola (BO)	-	RR	RR	ACG / ACG
Mp-230.06	16/05/19	Imola (BO)	-	RR	RR	ACG / ACG
Mp-230.07	16/05/19	Imola (BO)	-	SR	RR	ACG / CTG
Mp-230.08	16/05/19	Imola (BO)	-	RR	RR	ACG / ACG
Mp-230.09	16/05/19	Imola (BO)	-	SR	RR	ACG / CTG
Mp-230.10	16/05/19	Imola (BO)	-	SR	RR	ACG / CTG
Mp-230.11	16/05/19	Imola (BO)	-	RR	RR	ACG / ACG
Mp-230.12	16/05/19	Imola (BO)	-	RR	RR	ACG / ACG
Mp-231.01	16/05/19	Imola (BO)	-	SR	RR	ACG / TTG

Mp-231.02	16/05/19	Imola (BO)	-	RR	RR	ACG / ACG
Mp-231.03	16/05/19	Imola (BO)	+	SR	SR	ATG / ACG
Mp-231.04	16/05/19	Imola (BO)	-	SR	RR	ACG / TTG
Mp-231.05	16/05/19	Imola (BO)	-	SR	RR	ACG / TTG
Mp-231.06	16/05/19	Imola (BO)	-	SR	RR	ACG / TTG
Mp-231.07	16/05/19	Imola (BO)	-	SR	RR	ACG / TTG
Mp-231.08	16/05/19	Imola (BO)	-	SR	RR	ACG / TTG
Mp-231.09	16/05/19	Imola (BO)	-	SR	RR	ACG / TTG
Mp-231.10	16/05/19	Imola (BO)	-	SS	RR	CTG / TTG
Mp-231.11	16/05/19	Imola (BO)	-	SR	RR	ACG / TTG
Mp-231.12	16/05/19	Imola (BO)	-	SR	RR	ACG / TTG
Mp-232.01	01/06/19	Cesena (FC)	-	RR	SR	ATG / ACG
Mp-232.02	01/06/19	Cesena (FC)	-	RR	RR	ACG / ACG
Mp-232.03	01/06/19	Cesena (FC)	-	RR	SR	ATG / ACG
Mp-232.04	01/06/19	Cesena (FC)	-	RR	SR	ATG / ACG
Mp-233.01	01/06/19	Reda (RA)	-	RR	RR	ACG / ACG
Mp-233.02	01/06/19	Reda (RA)	-	RR	RR	ACG / ACG
Mp-233.03	01/06/19	Reda (RA)	-	RR	RR	ACG / ACG
Mp-233.04	01/06/19	Reda (RA)	-	RR	RR	ACG / ACG
Mp-234.01	01/06/19	Reda (RA)	-	RR	RR	ACG / ACG
Mp-234.02	01/06/19	Reda (RA)	-	RR	RR	ACG / ACG
Mp-234.03	01/06/19	Reda (RA)	-	RR	RR	ACG / ACG
Mp-234.04	01/06/19	Reda (RA)	-	RR	RR	ACG / ACG
Mp-235.01	08/05/19	Cesena (FC)	-	RR	RR	ACG / ACG
Mp-235.02	08/05/19	Cesena (FC)	-	RR	RR	ACG / ACG
Mp-235.03	08/05/19	Cesena (FC)	-	RR	RR	ACG / ACG
Mp-235.04	08/05/19	Cesena (FC)	-	RR	RR	ACG / ACG
Mp-236.01	08/05/19	Forli (FC)	+	SS	SR	ATG / CTG
Mp-236.02	08/05/19	Forli (FC)	+	SS	SR	ATG / CTG
Mp-236.03	08/05/19	Forli (FC)	-	SR	RR	ACG / CTG
Mp-236.04	08/05/19	Forli (FC)	-	SR	RR	ACG / CTG
Mp-237.01	22/05/19	Dovadola (FC)	-	SS	SS	ATG / ATG
Mp-237.02	22/05/19	Dovadola (FC)	-	SS	SS	ATG / ATG
Mp-237.03	22/05/19	Dovadola (FC)	-	SS	SS	ATG / ATG
Mp-237.04	22/05/19	Dovadola (FC)	-	SS	SS	ATG / ATG
Mp-238.01	20/05/19	Cesena (FC)	-	SR	RR	ACG / TTG
Mp-238.02	20/05/19	Cesena (FC)	-	SR	RR	ACG / TTG
Mp-238.03	20/05/19	Cesena (FC)	-	SR	RR	ACG / TTG
Mp-238.04	20/05/19	Cesena (FC)	-	SR	RR	ACG / TTG
Mp-239.01	23/05/19	Cesena (FC)	-	SS	SS	ATG / ATG
Mp-239.02	23/05/19	Cesena (FC)	-	SS	SS	ATG / ATG
Mp-239.03	23/05/19	Cesena (FC)	-	SS	SS	ATG / ATG
Mp-239.04	23/05/19	Cesena (FC)	-	SS	SS	ATG / ATG
Mp-240.01	24/05/19	Mondragone (CE)	-	SS	RR	ATG / CTG
Mp-240.02	24/05/19	Mondragone (CE)	-	SS	RR	ATG / CTG
Mp-240.03	24/05/19	Mondragone (CE)	-	SS	RR	ATG / CTG
Mp-240.04	24/05/19	Mondragone (CE)	-	SS	RR	ATG / CTG
Mp-241.01	22/05/19	Meldola (FC)	-	RR	RR	ACG / ACG
Mp-241.02	22/05/19	Meldola (FC)	-	SR	RR	ATG / ACG
Mp-241.03	22/05/19	Meldola (FC)	-	RR	RR	ACG / ACG
Mp-241.04	22/05/19	Meldola (FC)	-	RR	RR	ACG / ACG
Mp-242.01	22/05/19	Campomarino (CB)	-	SR	RR	ACG / CTG
Mp-242.02	22/05/19	Campomarino (CB	-	SR	RR	ACG / CTG
Mp-242.03	22/05/19	Campomarino (CB	-	SR	RR	ACG / CTG
Mp-242.04	22/05/19	Campomarino (CB	-	SR	RR	ACG / CTG

Dataset S1 (separate file). **Lists of genes differentially expressed (FDR < 0.05 and fold change > 2) between bifenthrin resistant (62H2, 88H2 and 92H6) and susceptible (1X and 4H) clones of *Myzus persicae* as determined by RNAseq analyses. Each of the 6 comparisons is shown in a new tab.**

Dataset S2 (separate file). **Maximum likelihood phylogeny of the amino-acid DDE transposase domain of the transposon identified in the VGSC subunit 1 gene of the *Myzus persicae* clones 62H2 and 88H2 with that of >1000 autonomous *Mutator*-like elements.**
