# Supplementary figures and images for: Transposon-mediated insertional mutagenesis unmasks recessive insecticide resistance in the aphid Myzus persicae
Source: Proc Natl Acad Sci U S A. 2021 May 31;118(23):e2100559118. doi: 10.1073/pnas.2100559118 (PMC8201860; doi:10.1073/pnas.2100559118)

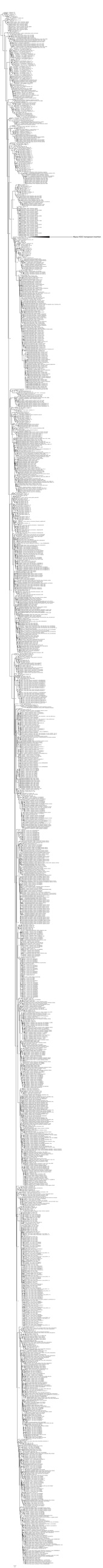

Supplement: Supplementary File [file pnas.2100559118.sd02.pdf]
